# Safety and Efficacy of the SHURUI Single-Port Robot-Assisted System via Transmesenteric Approach for Pyeloplasty in Upper Ureteral Stricture: A Single-Center Prospective Cohort Study

**DOI:** 10.1590/S1677-5538.IBJU.2026.0335

**Published:** 2026-06-18

**Authors:** Jiyu Yang, Bing Wang, Linsong Yang, Wenzhi Gao, Zihui Gao, Zhihua Li, Yaming Gu, Xuesong Li, Kunlin Yang

**Affiliations:** 1 Peking University Peking University First Hospital Department of Urology Beijing China Department of Urology, Peking University First Hospital, Institute of Urology, Peking University, National Urological Cancer Centre, Beijing, China; 2 Peking University First Hospital-Miyun Hospital Department of Urology Beijing China Department of Urology, Peking University First Hospital-Miyun Hospital, Beijing, China

## Abstract

**Purpose:**

Pyeloplasty is an established procedure for proximal ureteral strictures, with proven feasibility and favorable outcomes (1). The single-port robotic system offers potential advantages such as reduced invasiveness and improved cosmesis (2, 3), while the transmesenteric approach provides direct retroperitoneal access with minimal colonic mobilization (4, 5). However, the combination of single-port robotic platform with transmesenteric approach for pyeloplasty remains scarcely reported. This study aims to evaluate the safety and efficacy of this combined technique using the SHURUI single-port robotic surgical system.

**Materials and methods:**

From June 2024 to March 2025, 8 patients with upper ureteral stricture underwent robotic pyeloplasty via transmesenteric approach using the SHURUI single-port system. Demographic, perioperative, and follow-up data were prospectively collected. Clinical success was defined as symptom-free with no radiographic evidence of obstruction.

**Results:**

All surgeries were successfully completed without any conversion to laparoscopic or open procedures. The median docking time and console time were 4 (IQR: 2–8) min and 125 (IQR: 113–157) min, respectively. The median estimated blood loss was 10 (IQR: 10–10) ml, and none of the patients required intraoperative transfusion. The median postoperative stay was 4 (IQR: 3–6) days, with no complications occurring. With a median follow-up of 15 (IQR: 11–17) months, all patients achieved clinical success.

**Conclusions:**

The SHURUI single-port robotic system is safe and effective for pyeloplasty in upper ureteral stricture via transmesenteric approach. Further validation through multi-center studies with larger cohorts and longer-term follow-up is recommended.

**Available at:**VIDEO

## Data Availability

Data will be available upon request.
